# Protein acetylation regulates xylose metabolism during adaptation of *Saccharomyces cerevisiae*

**DOI:** 10.1186/s13068-021-02090-x

**Published:** 2021-12-17

**Authors:** Yong-Shui Tan, Li Wang, Ying-Ying Wang, Qi-En He, Zhi-Hua Liu, Zhen Zhu, Kai Song, Bing-Zhi Li, Ying-Jin Yuan

**Affiliations:** 1 Frontiers Science Center for Synthetic Biology and Key Laboratory of Systems Bioengineering (Ministry of Education), School of Chemical Engineering and Technology, Tianjin University, Tianjin, 300072 People’s Republic of China; 2 Synthetic Biology Research Platform, Collaborative Innovation Center of Chemical Science and Engineering (Tianjin), Tianjin University, Tianjin, 300072 People’s Republic of China; 3 Key Laboratory of MEMS of Ministry of Education, Southeast University, Nanjing, 210096 People’s Republic of China; 4 School of Chemical Engineering and Technology, Tianjin University, Tianjin, 300072 People’s Republic of China

**Keywords:** Xylose consumption memory, Protein acetylation, *Saccharomyces cerevisiae*, H4K5Ac, Synthetic biology

## Abstract

**Background:**

As the second most abundant polysaccharide in nature, hemicellulose can be degraded to xylose as the feedstock for bioconversion to fuels and chemicals. To enhance xylose conversion, the engineered *Saccharomyces cerevisiae* with xylose metabolic pathway is usually adapted with xylose as the carbon source in the laboratory. However, the mechanism under the adaptation phenomena of the engineered strain is still unclear.

**Results:**

In this study, xylose-utilizing *S. cerevisiae* was constructed and used for the adaptation study. It was found that xylose consumption rate increased 1.24-fold in the second incubation of the yYST12 strain in synthetic complete-xylose medium compared with the first incubation. The study figured out that it was observed at the single-cell level that the stagnation time for xylose utilization was reduced after adaptation with xylose medium in the microfluidic device. Such transient memory of xylose metabolism after adaptation with xylose medium, named “xylose consumption memory”, was observed in the strains with both xylose isomerase pathway and xylose reductase and xylitol dehydrogenase pathways. In further, the proteomic acetylation of the strains before and after adaptation was investigated, and it was revealed that H4K5 was one of the most differential acetylation sites related to xylose consumption memory of engineered *S. cerevisiae*. We tested 8 genes encoding acetylase or deacetylase, and it was found that the knockout of the *GCN5* and *HPA2* encoding acetylases enhanced the xylose consumption memory.

**Conclusions:**

The behavior of xylose consumption memory in engineered *S. cerevisiae* can be successfully induced with xylose in the adaptation. H4K5Ac and two genes of *GCN5* and *HPA2* are related to xylose consumption memory of engineered *S. cerevisiae* during adaptation. This study provides valuable insights into the xylose adaptation of engineered *S. cerevisiae*.

**Supplementary Information:**

The online version contains supplementary material available at 10.1186/s13068-021-02090-x.

## Background

Lignocellulosic biomass, such as energy crops, aquatic plants, forest biomass, and agricultural residues, is one of the most important renewable sources. Biofuels from lignocellulosic biomass has been considered as a good alternative to petroleum fuels due to the reduction of CO_2_ emission [1, 2]. The second generation bioethanol had been developed using lignocellulosic biomass to supply liquid fuel for vehicles [3–5]. Production of lignocellulosic bioethanol is mainly dependent on consists of lignocellulosic biomass [6–8]. Glucose and xylose are the major components in lignocellulosic hydrolysates produced from pretreatment and enzymatic hydrolysis. Glucose is a preferred substrate for ethanol production by *S. cerevisiae*, but xylose generally cannot be effectively converted to ethanol [9]. *S. cerevisiae* has engineering with xylose pathway to convert xylose to bioethanol [10, 11], and various strategies and genetic modifications have been developed to improve the xylose utilization of *S. cerevisiae* [12, 13]. The adaptation with xylose medium is still the most efficient approach to improve xylose utilization, but the mechanism of the adaptation is still unclear [14, 15].

In general, the microbial cells can store the information about current environment when encountering environmental changes. The stored information enables the strain to respond faster when returning to the original environment again [16, 17]. In the studies of galactose memory, the memory-induced the expression of *GAL* genes (*GAL1*, *GAL2*, *GAL7*, and *GAL10*) in *S. cerevisiae* could be activated much faster within 7 generations of the cell division after the strain was cultured on galactose medium once [18–20]. The expression levels of the galactose metabolic genes in *S. cerevisiae* changed along with the changes of the concentrations of glucose and galactose [21]. Heterokaryons formed by mating galactose-induced and un-induced *S. cerevisiae* also exhibited the cellular memory phenotype when the un-induced heterokaryons were placed in the galactose-induced cell cytoplasm, which revealed that the cytoplasmic in the galactose-induced cells contains some molecules that can induce the galactose memory [19]. Since microbes can store the memory in the form of molecular interactions, this phenomenon generally called transient memory [22].

Epigenetic regulation plays a critical role in facilitating the adaptation of microorganisms to fit the new environment [23]. There is close link between epigenetic and microbial memory. After a period of adaptation in the medium lacking inositol, the new generations of cells grow well on the medium lack of inositol [23]. It was found that the adaptation was related to maintaining the expression of *INO1* gene. The expression of the *INO1* gene is regulated by multiple factors, including the transcription factor SFL1, H2A.Z, and the methylation of histone H3 [24, 25]. During the adaption to the new environment, microbes may enter a protective state and exhibit stagnant or slow growth to regulate the gene expression [26]. Epigenetic modification could cause the regulation of gene expression to generate transient memory of the metabolic network [27–29]. Such transient memory should be very important for microbe adaptation. However, the mechanism under the transient memory is still unclear for xylose adaption of *S. cerevisiae*.

The present study aims to reveal the mechanism of transient memory of *S. cerevisiae* induced during adaptation. We first designed and constructed the xylose-utilizing *S. cerevisiae* with different xylose metabolic pathway. By switching culture between synthetic complete-glucose medium (SG) and synthetic complete-xylose medium (SX), we revealed the generation and erasure of the transient memory of xylose metabolism*.* Protein acetylation analysis indicated that acetylation of histone was involved in the process, and we figured out the key acetylation sites of histone and the key genes.

## Results

### The “xylose consumption memory” behavior of xylose-utilizing *S. cerevisiae*

**Fig. 1:**
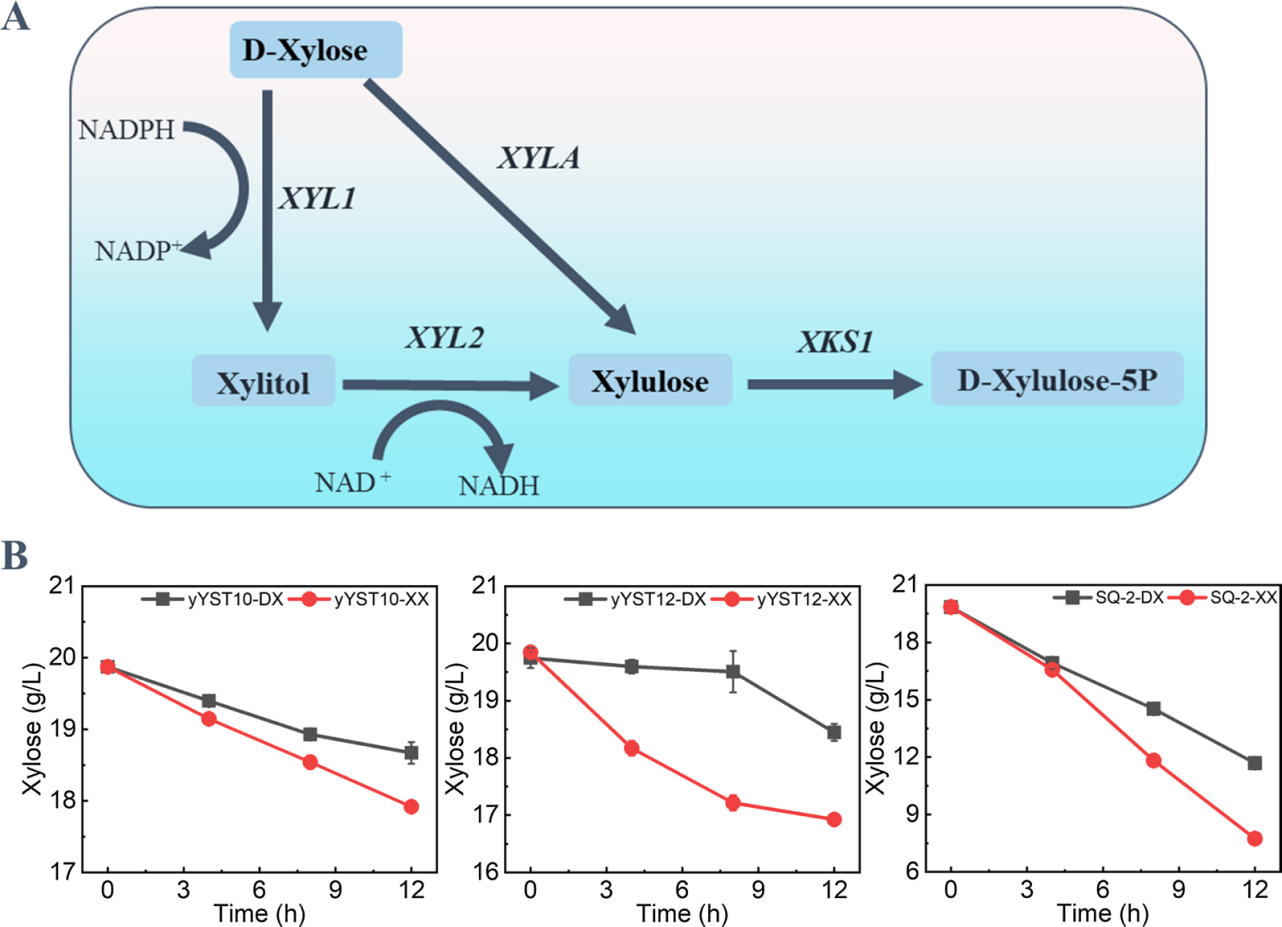
Construction of xylose-utilizing *S. cerevisiae* and their xylose consumption ability under different culture strategies in shake flask fermentation. **A** Xylose metabolic pathway constructed in *S. cerevisiae*. **B** Xylose consumption ability under different culture strategies, by which SQ-2, yYST10 and yYST12 were first cultured in synthetic complete-glucose medium (SG) or synthetic complete-xylose medium (SX) and then transferred to SX medium. The solid line represents the xylose consumption curve of the strain when “xylose consumption memory (XCM)” is not produced. The dashed line represents the xylose consumption curve of the strain when XCM is produced

The metabolic pathway with xylose reductase and xylitol dehydrogenase was engineered in yYST10 and yYST12, and SQ-2 with the same pathway is a gift from Professor Li-min Cao group at Capital Normal University (Fig. 1A, Table 1). To reveal the transient memory of xylose metabolism, we designed a transfer culture experiment for the engineered *S. cerevisiae* with xylose metabolic pathway. We determined the xylose consumption of the engineered *S. cerevisiae* during the first and the second incubations (Fig. 1B). The xylose consumption rate by the yYST12 strain was 0.108 g/L/h in the first incubation in SX medium, while the xylose consumption rate reached to 0.243 g/L/h in the second incubation in SX medium. Similar with yYST12, all these strains were able to consume xylose faster in second incubation in SX medium than that in the first one. We can use the xylose memory value as a metric to evaluate “xylose consumption memory (XCM)” of the engineered *S. cerevisiae* during the rapid adaptation. The xylose memory value of the yYST12 strain was 1.24, while it was 1.48 and 1.63 for the SQ-2 strain and the yYST10 strain, respectively. A larger memory value indicates high XCM of the strain in the rapid adaptation. All the xylose-utilizing *S. cerevisiae* used here exhibited the behavior of XCM during the rapid adaptation, but the XCM of these strains is different.

**Table 1:** *Saccharomyces cerevisiae* strains and plasmids used in this study

Strain	Alias	Description	Source	
SQ-2		MATα, *ura3*::*Ppgk1*-*XR4m*-*Tpgk1*/*Ppgk1*-XDH-*Tpgk1*/*Padh1*-*XK*-*Txks1*/*Padh1*-*RPE1*-*Trpe1*/*Ppgk1*-*RKI1*-*Tcyc1*/*Padh1*-*TAL1*-*Tadh1*/*Pkgd1*-*TAL1-Ttal1*/*Ppgk1*-*XYL1*-*Tadh1*/*Ppgk1*-*XYL2*-*Tpgk1*/*Ppgk1*-*XKS1*-*Tpgk1*		Capital Normal University
BY4742		MATα, his3Δ1, leu2Δ0, lys2Δ0, and ura3Δ0		This study
L2612		MATa, leu2-3, leu2-112, ura3-52, trp1-298 can1 cyn1 gal+		This study
yYST10		BY4742, (*ho::Ppgk1*-*XYL1-Tcyc1*-*Ptdh3*-*mXYL2-Ttef1*-*Tfba1*-*Ptpi1*-*XKS1*-*Txks1*)		This study
yYST12		L2612, (*ura3::Ppgk1-XYL1-Tpgk1-Ppgk1*-*mXYL2*-*Tpgk1*-*Ppgk1-XKS1-Txks1*)		This study
yYST24		BY4742, (pRS425-*Ptdh1*-*XKS1*-*Tpgk1*-*Ptdh3*-*RsXI*-*Tcyc1*)		This study
yYST31		BY4742, (*ho::Ptdh1-XKS1-Tpgk1-Ptdh3-RsXI-Tcyc1*)		This study
yYST210	H4K5R	yYST12, (*hhf1::HHF1*(*K5R*),*hhf1::HHF1*(*K5R*))		This study
yYST245	*ΔHDA1*	yYST12, (*ΔHDA1*)		This study
yYST246	*ΔHOS2*	yYST12, (*ΔHOS2*)		This study
yYST247	*ΔHST1*	yYST12, (*ΔHST1*)		This study
yYST248	*ΔRPD3*	yYST12, (*ΔRPD3*)		This study
yYST249	*ΔELP3*	yYST12, (*ΔELP3*)		This study
yYST250	*ΔGCN5*	yYST12, (*ΔGCN5*)		This study
yYST251	*ΔHPA2*	yYST12, (*ΔHPA2*)		This study
yYST252	*ΔSAS3*	yYST12, (*ΔSAS3*)		This study

### Microfluidic culture observed the “xylose consumption memory” of *S. cerevisiae*

**Fig. 2:**
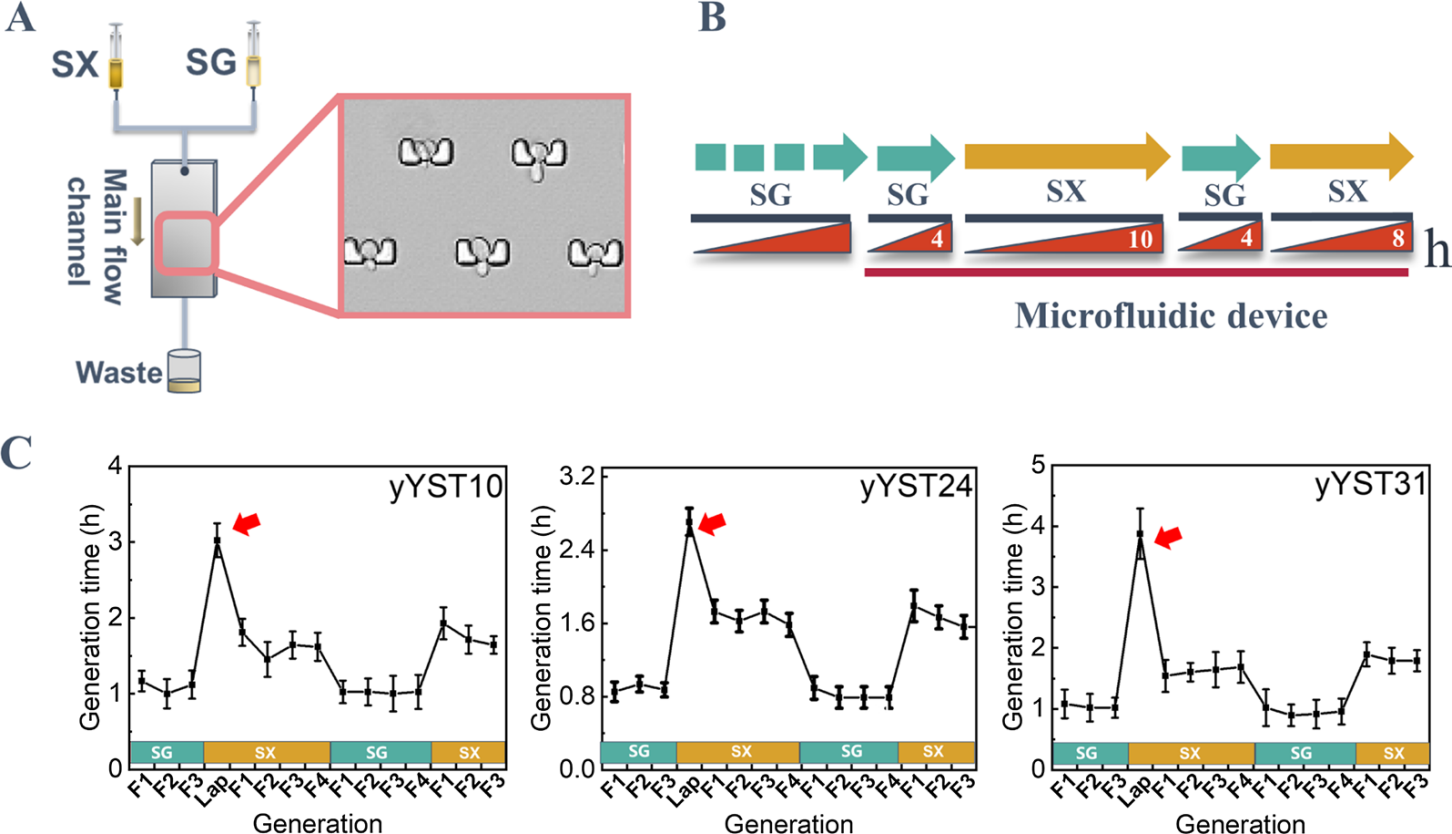
Microfluidic technology validated the “xylose consumption memory (XCM)” behaviors of *S. cerevisiae* in changing culture environments. **A** Schematic representation of the microfluidic device enabled free switching of culture medium and the micrograph of cells grown inside the growth chambers. **B** Schematic representation of the time course experiments to monitor the behavior changes of *S. cerevisiae* during carbon-source shifts. **C** The germination time of *S. cerevisiae* strains under different culture modes

We developed a microfluidic device with an array of single-cell traps for reliable immobilization of single yeast cells and long-term cell culturing under precisely controlled medium perfusion. The device also features rapid medium switching and microscopic monitoring of the in-situ XCM behavior at single cell level [30] (Fig. 2A). Initially, the strains of SQ-2, yYST10 and yYST12 were injected and cultivated in the microfluidic device for a test. However, the SQ-2 strain was unable to flow through the microfluidic device smoothly due to the larger cell size, while the yYST12 strain was unable to be dispersed into single cells due to the self-aggregation. Hence, we constructed another two strains (the yYST24, yYST31) with xylose isomerase pathway. Together with the strain of yYST10, the three strains were then cultured in the microfluidic device for the XCM study, respectively (Fig. 2B).

When the medium was first switched from SG medium to SX medium, the three strains immediately entered a delayed period, which exhibited slow growth state in budding. The duration of the delayed period varied from 2.5 h (h) to 5 h among all three strains. After the delayed period, the cells entered a recovery phase and gradually returned to the normal growth and division. After that, when the medium was switched back to SG medium, the strains rapidly recovered to the normal growth rate. And then, when the medium was switched to SX medium again, the second SX medium culture did not result in another severely delayed period in terms of the growth rate of all three strains. The duration of the delayed periods was much shorter than that in the first SX medium culture (Fig. 2C). Therefore, the microfluidic experiment further confirmed the XCM behaviors of the xylose-utilizing *S. cerevisiae* at single-cell level. These results indicated that the rapid adaptation of xylose metabolism for the engineered *S. cerevisiae* exactly existed under the switching of the media.

### Xylose induced histone acetylation

**Fig. 3:**
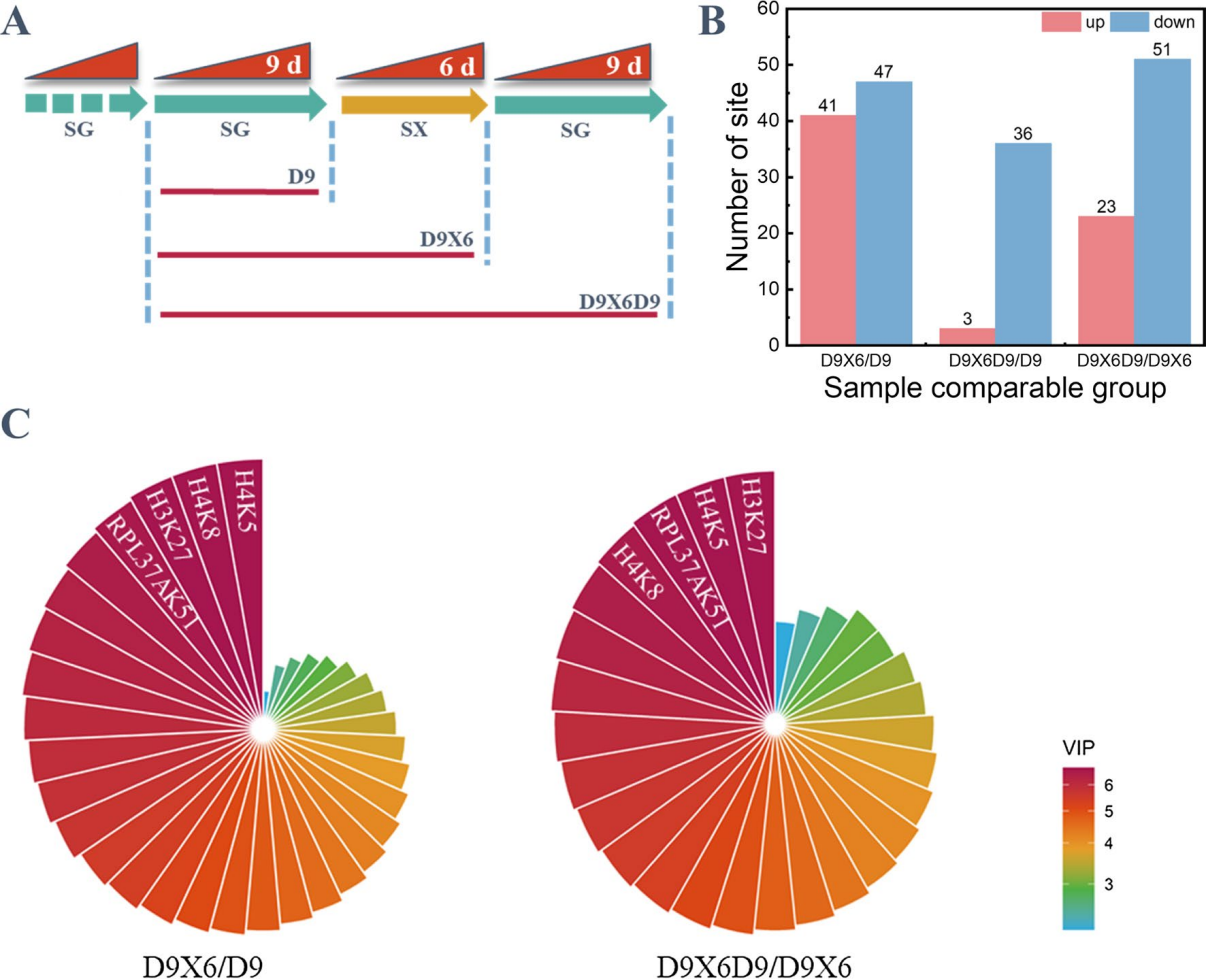
Identification of protein acetylation in *S. cerevisiae* under different culture modes. **A** Culture mode design and the cell sample acquisition flow chart. D9 indicates that the yYST12 strain cells was cultured in synthetic complete-glucose medium (SG) for 9 days (d) in continuous passages; D9X6 indicates that the yYST12 strain cells was cultured in SG medium for 9 days in continuous passages and then the cells was transferred to synthetic complete-xylose medium (SX) for 6 days in continuous passages; D9X6D9 indicates that the yYST12 strain cells was cultured in SG medium for 9 days, then the cells was transferred to SX medium for 6 days, and again the cells was transferred to SG medium for 9 days. **B** Comparative plot of lysine acetylation site changes in the yYST12 strain under different culture modes. **C** Least-squares method employed for analyzing the protein acetylation and variable importance index (VIP) plot according to the importance of the acetylation site

Epigenetic regulation changes to resisting the environmental stress in plants, and these changes could be inherited for several generations [31]. Histone acetylation is an important epigenetic modification. To analyze the potential epigenetic modification of *S. cerevisiae*, the culture strategy was further designed (Fig. 3A). We used yYST12 strain as an example for histone acylation analysis due to the significant XCM during the rapid adaptation. The samples for protein acylation analysis were collected at three points: no XCM was produced; XCM was produced; XCM was disappeared. In the protein acetylation analysis, 871 lysine acetylation sites in 420 protein groups were identified, among which 841 lysine acetylation sites in 403 proteins were quantified. The fold-change cutoff was set as more than 1.2 or less than 0.83 according to the quantitative ratios. Among these quantified sites, the acylation at 41 lysine sites was up-regulated, while 47 lysine sites down-regulated in D9X6 vs. D9. In addition, the acylation at 3 sites was up-regulated, while 36 sites down-regulated in D9X6D9 vs. D9. The acylation at 23 lysine sites was up-regulated, while 51 sites down-regulated in D9X6D9 vs. D9X6 (Fig. 3B).

A partial least squares deep regression method was employed to analyze the data of the protein acetylation. Significant acetylation sites were identified using the partial least squares depth regression algorithm. After removing the acetylation sites with missing values, 324 acetylation sites (variables) remained in the acetylation group. Combined with the variables importance on projection (VIP) analysis, all variables were arranged in descending order of importance (Additional file 3). The greater the VIP rating, the more important the acetylation site was to the XCM. In the comparison of D9X6 vs. D9, it was found that the VIP values of the acetylation sites of histones H4 lysine 5 (H4K5), histones H4 lysine 8 (H4K8), histones H3 lysine 27 (H3K27), and histones H4 lysine 16 (H4K16) were 1.3136, 1.3135, 1.3122, and 1.3111, respectively, which were the top four VIPs. In the comparison of D9X6 vs. D9X6D9, the top 4 VIP values were at the acetylation sites of H4K5, H4K8, H3K27, and protein RPL37A lysine 51 (RPL37AK51), and they were 1.3529, 1.3527, 1.3514 and 1.3511, respectively. These results indicated that H4K5, H4K8 and H3K27 may play an important role in the generation of XCM (Fig. 3C).

### H4K5 acetylation is related to the XCM

**Fig. 4:**
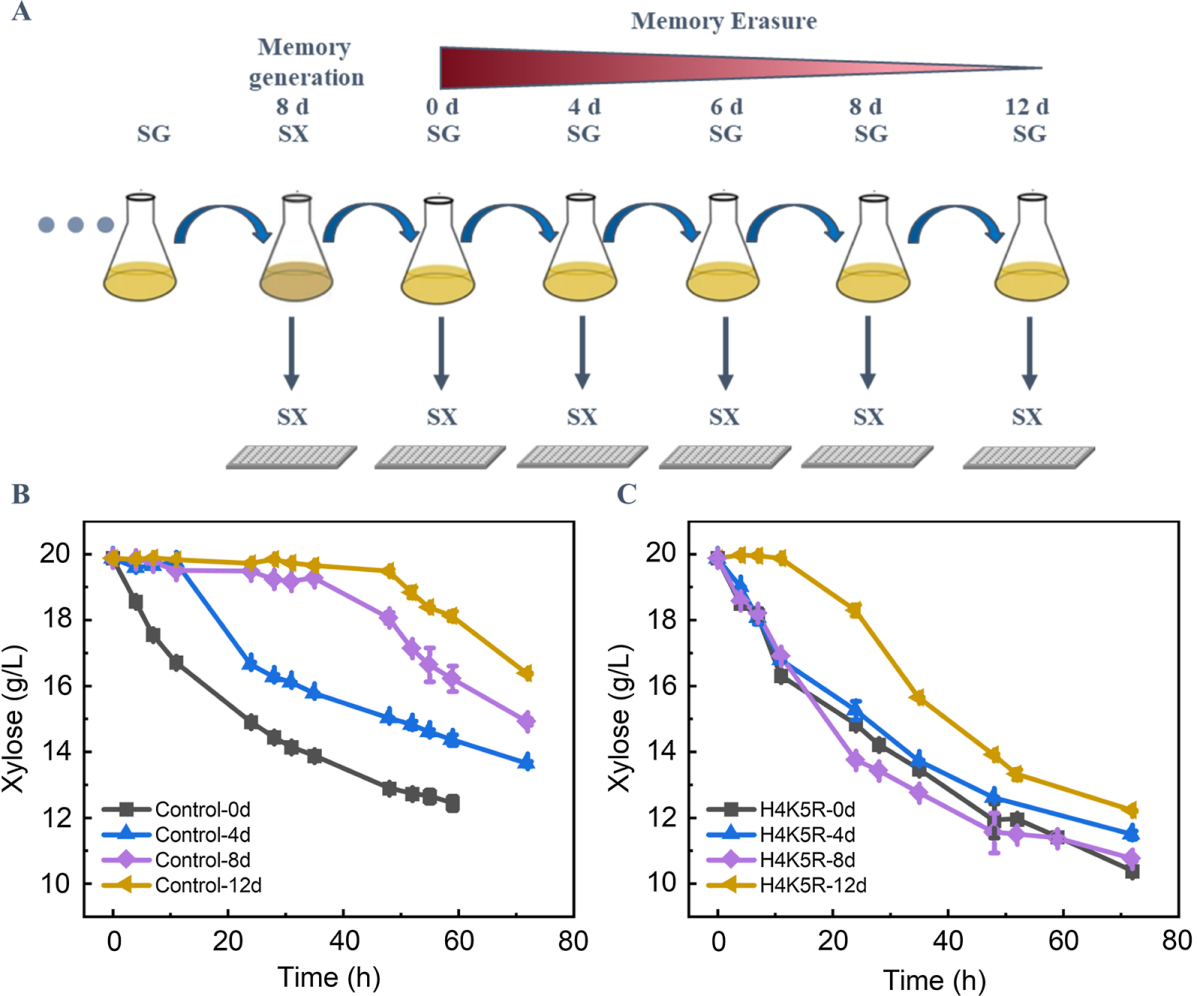
Function loss of lysine acetylation site function regulated the “xylose consumption memory (XCM)” behaviors of *S. cerevisiae*. **A** Culture mode design induced the forgetting of XCM. The yYST210 (H4K5R) and control yYST12 strains were continuously incubated in synthetic complete-xylose medium (SX) to induce the XCM. The strains were transferred to synthetic complete-glucose medium (SG) and cultivated for 0–12 days (d), respectively, and then finally transferred to SX medium. **B,** **C** Xylose consumption ability of the strains after the acetylation capacity loss at the acetylation sites of control and H4K5R strains

Several key protein acetylation sites have been identified in the yYST12 strain by proteomic analysis. It is necessary to further evaluate the specific correlations between these protein acetylation sites and the XCM behavior of the engineered *S. cerevisiae*. We mutated the lysine to arginine at the most significant acetylation site of histone, H4K5, and so the acetylation was not able to occur at H4K5. The erasure of XCM of yYST210 (H4K5R) strains was evaluated as designed in Fig. 4A. The XCM of the control strain yYST12 was erased step by step from 2 days (d) to 12 days after moved to SG medium, while the XCM of the yYST210 strain was stable until 8 days after moved to SG media (Fig. 4B, C). The XCM of the yYST210 strain was start to be erased from 8 to 12 days after moved to SG medium. These results indicated that the acylation of H4K5 is very important for XCM of the engineered *S. cerevisiae*. Forgetting values according to the xylose consumption rates were calculated to evaluate the XCM ability of the engineered *S. cerevisiae*, and the higher of the forgetting values represent faster rates of XCM erasure. The forgetting values of the YST210 and control strains yYST12 were 0.96 and 3.89 according to the xylose consumption performance at 8 days after moved to SG medium (Additional file 1: Fig. S1). The results indicated that the loss of H4K5 acylation significantly decreased the erasure rate of XCM of the engineered *S. cerevisiae*.

### Effect of enzymes related to acetylation modification of histone on XCM

**Fig. 5:**
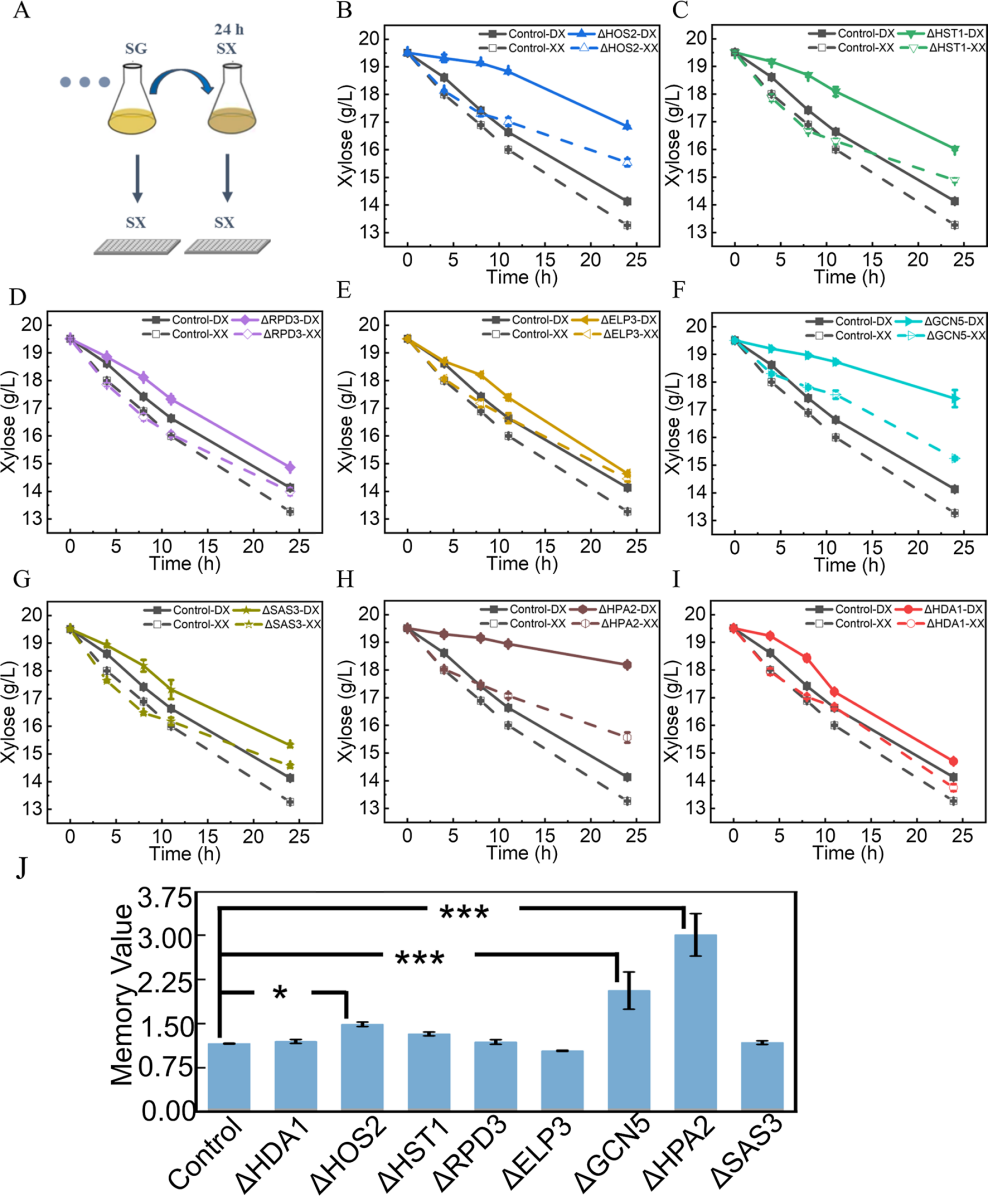
Deletion of a single acetylation modifying enzyme regulated the “xylose consumption memory (XCM)” behaviors of *S. cerevisiae*. **A** Culture mode induced the XCM of *S. cerevisiae*. Xylose consumption was detected by transferring the cells to synthetic complete-xylose medium (SX) after incubation in synthetic complete-glucose medium (SG). Then xylose consumption weight was detected by transferring the strain to SX medium after incubation in SX medium for 24 h (h). **B**–**I** are the knockdown of deacetylase and acetylase regulated the xylose consumption weight. The solid line represents the xylose consumption curve of the strain when XCM is not produced. The dashed line represents the xylose consumption curve of the strain when XCM is produced. **J** is the memory value of the acetylation-related enzyme knockdown assay. Larger memory values indicate that *Saccharomyces cerevisiae* is more prone to produce XCM. *P < 0.05, ***P < 0.001

Aforementioned results indicated that the modification of histone acetylation site has the influence on the XCM of *S. cerevisiae*. However, the specific enzymes related to XCM were still unknown. We selected the representative acetylases (ELP3, GCN5, SAS3, and HPA2) and deacetylases (HDA1, HOS2, HST1, and RPD3) for further study [32]. We constructed the strains with single gene deletion for the 8 enzymes, and the effects of the gene deletion on XCM were evaluated. The xylose consumption performance for each engineered strain was analyzed before and after the generation of XCM. The knockout of these genes reduced the xylose consumption rate in the engineered *S. cerevisiae* (Fig. 5B–I). The memory value of the control yYST12 strain was 1.16. After knocking out *HOS2* gene, the memory value of the yYST246 strain (*ΔHOS2*) increased to 1.49. For the deletion of *GCN5* or *HPA2*, the xylose consumption of the yYST250 strain (*ΔGCN5*) and the yYST251 strain (*ΔHPA2*) was significantly reduced compared with that of the control yYST12 strain. After 24 h incubation in SX medium, the xylose consumption of the *ΔGCN5* and *ΔHPA2* strains was significantly accelerated. The memory values of the *ΔGCN5* and *ΔHPA2* strains increased to 2.06 and 3.00, respectively, while knock-out of other acetylation-related genes had no significant effect on the memory value (Fig. 5J). All these results suggested the representative acetylases (GCN5, HPA2) were the key enzymes to enhance the XCM behavior of engineered *S. cerevisiae* compared with other acetylases and deacetylases selected in the present study.

## Discussion

Environmental variations are the critical challenges for microbes, and microbes usually regulates and adapt the gene expression to fit the variations. Therefore, the rapid adaptation is very important for microbes to overcome the environmental variations. If the variations occur repetitively, microbes could generate cellular memory for the gene expression to fit the environmental variations [33]. The cellular memory could endow the microbes the rapid adaptation ability to fit the environmental variations, and could pass to offspring for several generations [34]. Due to resisting the environmental variations, yeast could change the epigenetic states, such as the modification of chromosomes, and the chromatin structures beside the gene promoters [20, 35]. When the yeast strain was exposed to galactose, yeast generated the cellular memory of the active transcriptional state for *GAL* gene [20, 36]. Before the chromosome structure recovered back to the inactive state, the re-addition of galactose induced the expression of *GAL* gene much more rapidly [37, 38]. When microbes adapt the gene expression according to the environmental variation, microbial growth and metabolism usually slows down until the fitness is achieved for the new environment. As we observed in this study, the division time of engineered yeast was much longer when they were moved to xylose for the first time. Similar performance was also observed for *E. coli*. When *E. coli* was moved to galactose for the first time, the growth decreased. The cellular memory enabled *E. coli* to adapt much rapidly for galactose at the second time, and the cellular memory about galactose metabolism could maintain for several generations in *E. coli* [39]. Besides carbon source switching, microbial cellular memory was also observed during the adaptation to other stress. A short pre-culture of yeast in the condition with high NaCl concentration significantly increased the resistance to salt stress, and much more rapid adaptation to salt stress was observed during the re-exposure to NaCl [40]. Cellular memory of yeast was also reported in the response of the short hyperosmotic stress with high concentration of sorbitol [41]. In this study, we reported the cellular memory of xylose metabolism when engineered yeast was moved to xylose from glucose, and the cellular memory helped yeast to fit xylose metabolism more rapidly.

For the unicellular organisms, the cellular memory for the environmental fluctuations passed to the daughter cells by epigenetic inheritance, including modification of histone, regulation of chromatin structure, regulation of transcriptional factors and so on [42]. Histone methylation has been revealed to be related to cellular memory in yeast and other organisms [43]. Histone modifications might serve as the basis for epigenetic memory [44]. In *S. cerevisiae*, histone modifications include methylation, acetylation, phosphorylation and so on. In addition, there are several cites on histone for the modifications. Here we reported that the acetylation of H4K5 was closely related to XCM. We also figured out that *GCN5* and *HPA2* encode the key enzymes for modification at H4K5 to regulate XCM of engineered yeast. GCN5 histone acetyltransferase was previously revealed in transient stress-induced reprogramming of the genome [45]. *HPA2* encodes the acetyltransferase, similar to *GCN5*, *HAT1*, *ELP3*, and *HPA3*, which can acetylate histones H3 and H4 [46]. Recently, it was revealed that H4 histone acetyltransferases, only worked during the transcription process in yeast [47]. Combined with our results, it is convincing that the acetylation of histone is very important part for maintaining cellular memory for previous metabolic states of yeast.

## Conclusions

In the present study, the XCM in *S. cerevisiae* has been induced with a switch of culture modes, more important, the molecular mechanisms associated with XCM had been systematically elucidated from a protein acetylation perspective. Results indicated that protein acetylation can regulate multigene pathways, which in turn affected the XCM of *S. cerevisiae*. The multiple technologies had been employed to identify the acetylation sites and alter the acetylation modifying-related enzymes, and confirmed that acetylation modifications significantly affected the ability of XCM in *S. cerevisiae*. A systematic exploration of the protein acetylation regulation in *S. cerevisiae* provides valuable insights into the microbial adaptation to complex industrial environments.

## Methods

### Strains and plasmids

All primers were synthesized by Genewiz (China) and listed in Additional file 1: Table S1. *S. cerevisiae* L2612 (MATa, leu2-3, leu2-112, ura3-52, and trp1-298 can1 cyn1 gal+), BY4742 (MATα, his3Δ1, leu2Δ0, lys2Δ0, and ura3Δ0) were used for constructing recombinant strains. *E. coli* Top10 was purchased from Beijing Biomedical Co., Ltd, and used for gene cloning and plasmid construction. The required promoters and terminators were amplified from *S. cerevisiae* BY4742 strain. The required genes were synthesized by GenScript China Inc. and then assembled using Vazyme’ ClonExpress MultiS One Step Cloning Kit (C113). In addition, we have constructed some engineered strains. The yYST24 strain have multicopy genes encoding xylose metabolic pathway, while yYST31 and yYST10 strains have the single copy. The SQ-2, yYST10 and yYST12 strains were engineered with the xylose reductase (XR) and xylitol dehydrogenase (XDH) pathway, while the other strains were engineered with the xylose isomerase (XI) pathway. The yYST24, yYST31 and yYST10 strains were constructed based on BY4742 chassis.

Design of the donor DNA for introduction of point mutation and knockout using the CRISPR/Cas9 system [48]. The protospacer adjacent motif (PAM) sequences of guide RNAs (gRNAs) were designed using E-CRISP Design (http://www.e-crisp.org/E-CRISP/designcrispr.html) [49]. The sequences of gRNAs used in this study are summarized in Additional file 1: Table S2. The detailed information of engineered *S. cerevisiae* strains and plasmids used in this study had been provided in Table 1.

### Media preparation and culture conditions

SG medium consists of 6.7 g/L yeast nitrogen base without amino acids, 20 g/L glucose, 0.1 g/L leucine, 0.02 g/L histidine, 0.02 g/L uracil and 0.02 g/L tryptophan. SX medium consists of 6.7 g/L yeast nitrogen base without amino acids, 20 g/L xylose, 0.1 g/L leucine, 0.02 g/L histidine, 0.02 g/L uracil and 0.02 g/L tryptophan. SC-Ura medium was prepared from 6.7 g/L yeast nitrogen base without amino acids, 20 g/L glucose, 0.1 g/L leucine, 0.02 g/L histidine, and 0.02 g/L tryptophan. In the experiment of microfluidic culture, SG medium and SX medium were filtered through a 0.2-µm filter and polyethylene–polypropylene glycol were added to make a final concentration of 0.5%. *E. coli* was cultivated on the luria–bertani medium (10 g/L peptone, 5 g/L yeast extract, and 5 g/L sodium chloride) with 100 mg/L ampicillin.

### Xylose consumption experiment

To evaluate the potential behaviors of *S. cerevisiae* with the switch of medium, the strains were first cultured in SG medium for 24 h and then transferred to SX medium and cultured for 24 h. The xylose content was determined by high-performance liquid chromatography (HPLC). After that, these strains were transferred to SX medium and cultured for another 24 h. The xylose content was determined by HPLC. Fermentation experiments were performed in 250 mL triangular flasks at 30 ℃ and 200 rpm with a working volume of 100 mL media.

### Microfluidic culturing and testing

A microfluidic channel integrated with Yrot traps was employed in this study [50]. In this experiment, the microchannel was first sterilized using 75% ethanol injected from the inlet and incubated at 100 ℃ overnight for residual liquid evaporation. The cell suspension was then injected into the channel at a flow rate of 5 µL/min for 5 min, and then the inlet was switched to fresh SG medium for long-term culturing at a flow rate of 10 µL/min. After 4 h of culturing and adaption of loaded cells, the SG medium was then switched to SX medium. After 10 h of culturing in SX medium, the flowing medium in the main channel was switched back to SG medium again for 4 h. Finally, the flowing medium in the main channel was switched to SX medium again for 6 h (Fig. 2B).

During the experiments, the microfluidic chip was fixed by a customized holder, with its inlet and outlet connected to a glass syringe and a waste collection, respectively. The whole system was together kept on an inverted confocal microscope (FV3000, Olympus Co., Japan) and then imaged using a 20× objective lens (UCPLFLN, 0.7 NA, Olympus Co., Japan, correction ring adjusted to 0.5 mM). Bright-field images were automatically scanned at an interval of 10 min using a software workflow (FV31S-SW, Olympus Co., Japan). A Z-axis Drift Compensation system (IX3-ZDC2, Olympus Co., Japan) helped to ensure the sharp focusing of samples throughout the long-term monitoring.

### Protein acetylation analysis

The yYST12 strain was first prepared in SG medium and the medium was refreshed every 24 h. The yYST12 strain was incubated in SG medium for a total of 9 days and labeled as D9. The yYST12 strain after culture in SG medium for 9 days did not have a behavior of xylose consumption memory. The yYST12 strain was then transferred to SX medium for a total of 6 days and the medium was refreshed every 48 h. The yYST12 strain under this culture condition was labeled as D9X6, which have a behavior of xylose consumption memory. The yYST12 strain was finally transferred to SG medium for a total of 9 days and the medium was refreshed every 24 h. The yYST12 strain under this culture condition was labeled D9X6D9, which shows the disappearance of xylose consumption memory. The fermentation was carried out at 30 ℃ and 200 rpm. Cells at the middle of the logarithmic growth cycle were harvested. The approaches involving TMT labeling, HPLC fractionation, Kac antibody affinity enrichment, and LC–MS/MS were employed to quantify the dynamic changes in the whole acetylome. The quantitative ratio of > 1.2 indicated the upregulation of sites, whereas the quantitative ratio of < 0.83 was indicated the downregulation of sites. To further understand the function and feature of the identified and quantified proteins, gene annotation was performed based on different categories, such as gene ontology (GO), domain, pathway, and subcellular localization. Both the identified and quantifiable proteins were annotated. Comparison group-based clustering was performed for D9X6 vs. D9, D9X6D9 vs. D9, and D9X6D9 vs. D9X6 groups. Bioinformatics analyses such as GO annotation, domain annotation, subcellular localization, kyoto encyclopedia of genes and genomes pathway annotation, and functional cluster analysis were performed to annotate the quantifiable lysine-acetylated targets in response to drug treatment.

Based on the results, further studies, such as XCM-related acetylation site prediction. Before training the data set, the assay data were preprocessed. Partial least square (PLS) is an efficient statistical classification technique suitable for analyzing high-dimensional data and genomic and proteomic data, especially for the problems of classification and dimension reduction in bioinformatics and genomics [51]. PLS is a commonly used feature extraction algorithm. This algorithm is based on the idea of latent variables that model the relationship between the input variable Xn × *m* (*n*: loci, *m*: samples) and the response variable Y1 × *m*. For example, in the case of D9 vs. D9X6, Y is a column vector, such as [1, 1, 1, –1, –1, –1], wherein D9 corresponds to 1 and D9X6 corresponds to –1. For more convincing results, the VIP was explored to calculate the importance of each site to the response variable, which is the basis for selecting the signature sites [52]:

1 $${\text{VIP = }}\sqrt {p \times \left( {q{\text{/sum}}\left( s \right)} \right)} ,$$

where *p* is the number of genes in the training data set, and

2 $$s{\text{ = diag}}\left( {\user2{T^{\prime}} \times T \times Q \times Q^{\prime}} \right)$$

3 $$q\,{ = }\,\user2{s^{\prime}} \times w,$$

where the parameters ***T***, ***Q***, and ***w*** are calculated using PLS; ***w*** is the unitized form of ***W***.

### Fermentation experiments to lose XCM

To study the xylose consumption performance, the strains were first cultured in SX medium for 6 days, and then transferred to SG medium and cultured for 0, 8, 12, 16, and 20 days, respectively. After that, these strains were again transferred to SX medium for high-throughput fermentation experiments. For high-throughput fermentation experiments, xylose consumption was analyzed and used as a metric for assessing the XCM. The SG medium was refreshed every 24 h, while the SX medium was refreshed every 48 h in these experiments (Fig. 4A). These strains were performed in 250 mL triangular flasks at 30 ℃ and 200 rpm with a working volume of 100 mL. High-throughput fermentation experiments were performed using 96-well plates at 30 ℃ and 900 rpm with a working volume of 230 µL.

### Fermentation experiments to generate XCM

To study the production of XCM, the yYST245, yYST246, yYST247, yYST248, yYST249, yYST250, yYST252 and yYST12 were first incubated in SG medium for 8 days and then transferred to SX medium for 24 h. At the same time, these strains were transferred to SX medium for high-throughput fermentation experiments. After that, these strains were again transferred to SX medium for high-throughput fermentation experiments. For high-throughput fermentation experiments, xylose consumption was analyzed and used as a metric for assessing the XCM (Fig. 5A). These strains were performed in 250 mL triangular flasks at 30 ℃ and 200 rpm with a working volume of 100 mL. High-throughput fermentation experiments were performed using 96-well plates at 30 ℃ and 900 rpm with a working volume of 230 µL.

### Stagnation rate, forgetting value and memory value

*Saccharomyces cerevisiae* was cultured in microfluidic, according to the first and second delay period, and the stagnation rate is obtained. A higher value of this value indicates that the strain has a better capacity of xylose consumption memory:

4 $${\text{Stagnation rate = }}\frac{{\varvec{A}}}{{\varvec{B}}},$$

where*** A*** is cell stagnation growth time during the first SX medium culture in microfluidic, ***B*** is cell stagnation growth time during the second SX medium culture in microfluidic.

Calculation of forgetting values for strains losing xylose consumption memory:

5 $${\text{Forgetting value = }}\frac{{\varvec{C}}}{{\varvec{D}}},$$

where ***C*** represents the weight of xylose consumption that *S. cerevisiae* were cultured in SX medium continuously for 6 days and transferred to SX medium for 48 h. ***D*** represents the weight of xylose consumption that *S. cerevisiae* were cultured in SX medium continuously for 6 days, then transferred to SG medium for 8 days, and finally transferred to SX medium for 48 h.

Compare the xylose consumption weight of strains after the xylose memory 24 h with strains before the xylose memory is generated:

6 $${\text{Memory value = }}\frac{{\varvec{E}}}{{\varvec{F}}},$$

where ***E*** represents the weight of xylose consumption that *S. cerevisiae* were continuously incubated in SG medium for 8 days, then transferred to SX medium for 24 h, and finally transferred to SX medium for 24 h. ***F*** represents the weight of xylose consumption that *S. cerevisiae* were cultured in SG medium continuously for 8 days and transferred to SX for 24 h.

### Xylose determination

Medium samples before and after fermentation were filtered through 0.2 µm filters before injection into the HPLC system. HPLC (Waters e2695/2414) consists of an Aminex HPX-87H ion-exchange column (Bio-Rad, Hercules, USA) using 0.5 mM H2SO4 as mobile phase at a flow rate of 0.6 mL/min with the column temperature of 65 ℃ [15].

## Supplementary Information

**Additional file 1: Table S1.** Primers used in this work. **Table S2.** gRNAs used in this work. **Fig. S1.** Comparison of forgetting value between strain yYST210 (H4K5R) and control yYST12. The H4K5R and control yYST12 strains were incubated in synthetic complete-xylose medium (SX) continuously to induce XCM, and the strains were then transferred to synthetic complete-glucose medium (SG) for 0 and 8 days, respectively, and finally incubated in SX medium for 48 h (h). ***P < 0.001.

**Additional file 2:** Data related to protein acetylation.

**Additional file 3:** Variables Importance on Projection.

## Data Availability

The data sets for this study are included in this published article and its Additional files 1, 2 and 3.

## Abbreviations

*S. cerevisiae*: *Saccharomyces cerevisiae*
XCM: Xylose Consumption Memory
PLS: Partial Least Squares
*E. coli*: *Escherichia coli*
SG: Synthetic Complete-glucose Medium
SC-Ura: Synthetic Complete-glucose Medium Lacking Uracil
SX: Synthetic Complete-xylose Medium
TMT: Tandem Mass Tags
LC–MS/MS: Liquid Chromatography–tandem Mass Spectrometry
HPLC: High-performance Liquid Chromatography
PAM: Protospacer Adjacent Motif
gRNAs: Guide RNAs
CRISPR: Clustered Regularly Interspaced Short Palindromic Repeat
