# Supplementary material for: Protein acetylation regulates xylose metabolism during adaptation of Saccharomyces cerevisiae
Source: Biotechnol Biofuels. 2021 Dec 17;14:241. doi: 10.1186/s13068-021-02090-x (PMC8684234; doi:10.1186/s13068-021-02090-x)
Supplement: Supplementary file 1 — Additional file 1: Table S1. Primers used in this work. Table S2. gRNAs used in this work. Fig. S1. Comparison of forgetting value between strain yYST210 (H4K5R) and control yYST12. The H4K5R and control yYST12 strains were incubated in synthetic complete-xylose medium (SX) continuously to induce XCM, and the strains were then transferred to synthetic complete-glucose medium (SG) for 0 and 8 days, respectively, and finally incubated in SX medium for 48 h (h). ***P < 0.001. [file 13068_2021_2090_MOESM1_ESM.docx]

Additional file 1

**Protein acetylation regulates xylose metabolism during adaptation of *Saccharomyces cerevisiae***

Yong-Shui Tan ^a,b^, Li Wang ^a,b^, Ying-Ying Wang ^c^, Qi-En He ^d^, Zhi-Hua Liu ^a,b^, Zhen Zhu ^c^ *, Kai Song ^d^ *, Bing-Zhi Li ^a,b^ *, Ying-Jin Yuan ^a,b^

^a^ Frontiers Science Center for Synthetic Biology and Key Laboratory of Systems Bioengineering (Ministry of Education), School of Chemical Engineering and Technology, Tianjin University, Tianjin, 300072, PR China

^b^ Synthetic Biology Research Platform, Collaborative Innovation Center of Chemical Science and Engineering (Tianjin), Tianjin University, Tianjin, 300072, PR China

^c^ Key Laboratory of MEMS of Ministry of Education, Southeast University, Nanjing, 210096, PR. China

^d^ School of Chemical Engineering and Technology, Tianjin University, Tianjin, 300072, PR China

*Corresponding author: Bing-Zhi Li, [bzli@tju.edu.cn](mailto:bzli@tju.edu.cn); Zhen Zhu, zhuzhen@seu.edu.cn; Kai Song, ksong@tju.edu.cn

**Additional file 1**

**Table S1 Primers used in this work**

| Primer ID | Sequence (5’-3’) |  |
| --- | --- | --- |
| g-PGK1t-R | aatgataaactcgaactgaaaaagcgtgttaacgaacgcagaattttcgagttattaaac |  |
| g-TDH3p-F | gtttaataactcgaaaattctgcgttcgttaacacgctttttcagttcgagtttatcatt |  |
| g-delta-L-F | TAGGGCGAATTGGGTACCGGGCCCCCCCTCGAGTGAGAAATGGGTGAATGTTGAGATAA |  |
| g-CYC1t-R | CAGATTCCCTTTTATGGATTCCTAAATCCTaaagccttcgagcgtcccaaaac |  |
| g-delta-R-F | gttttgggacgctcgaaggctttAGGATTTAGGAATCCATAAAAGGGAATCTG |  |
| delta-R-R | GTGGCGGCCGCTCTAGAACTAGTGGATCCTGTTGGAATAGAAATCAACTATCATCTACT |  |
| OE-L1-1-Ho-R-F | GCTGTAAAAAGTATGGCTTGGATGC |  |
| OE-L1-2-HO-R-R | gtttaataactcgaaaattctgcgttcgttACAGTAGCTGACATACCAAGAGATCTT |  |
| OE-L1-3-PGK1t-F | AAGATCTCTTGGTATGTCAGCTACTGTaacgaacgcagaattttcgagttattaaac |  |
| OE-L1-4-XKS1-R | cgcgaggctaaatttccattgaaca |  |
| L2-1-XKS1-F | agctcatcactgaattttctttcacgt |  |
| L2-XylA-R | gaacctaagcaaacttccggtaagc |  |
| L3-TDH1p-F | tggatcagaagctcagcccaac |  |
| L3-TDH3p-R | ccttctattaccttctgctctctctgat |  |
| OE-L4-1-XylA-F | caacatcgtggaagcagtagtaagg |  |
| OE-L4-2-TDH3p-R | ATTGGACTTTTCTTCCTTCAATGATTTCCTaacacgctttttcagttcgagtttatc |  |
| OE-L4-3-HO-L-F | gataaactcgaactgaaaaagcgtgttAGGAAATCATTGAAGGAAGAAAAGTCCAAT |  |
| L4-4-HO-L-R | CACTTCACGTGCTTCTGGTACATAC |  |
| L5-RsXI-R | agttgctttgccaggttctggta |  |
| OE-L7-RsXI-F | ccaaacatatcttgaccagtagcacac |  |
| GS-HO-L-F | TCGAATTCCTGCAGCCCGGGGGATCCcttccatagcatctagcacatactcgat |  |
| GS-HO-L-R | AAGTCAGGAATCGCGGCCGCaggaaatcattgaaggaagaaaagtccaatg |  |
| GS-PGK1p-F | GCGGCCGCGATTCCTGACTTCAACTCAAGACGCA |  |
| GS-PGK1p-R | ttcaacttgatggatggcatTGTTTTATATTTGTTGTAAAAAGTAG |  |
| GS-XYL1-F | atgccatccatcaagttgaactct |  |
| GS-XYL1-R | tcattagacgaaaattgggatcttatccca |  |
| GS-CYC1t-F | tcccaattttcgtctaatgacatgtaattagttatgtcacgcttacattcac |  |
| GS-CYC1t-R | GCGGCCGCTCTAGAACTAGTGGATCCaaagccttcgagcgtccca |  |
| GS-mXYL2-F | AACACACATAAACAAACAAAatgactgctaacccatccttgg |  |
| GS-mXYL2-R | gttaagtgtttgattgatggtccagaatgataaaaataaggagattgataaga |  |
| GS-TEF1t-F | TCGAATTCCTGCAGCCCGGGggatccaaataaggagattgataagacttttctagttgcatatc |  |
| GS-TEF1t-R | ATCAATTAATTTGAATTAACgatagcgccgatcaaagtatttgttacga |  |
| GS-FBA1t-F | GTTAATTCAAATTAATTGATATAG |  |
| GS-FBA1t-R | GCGGCCGCTCTAGAACTAGTggatccAAAGATGAGCTAGGCTTTTGTAAAAATATC |  |
| GS-FT-F | TCGAATTCCTGCAGCCCGGGGGATCCGTTAATTCAAATTAATTGATATAG |  |
| GS-FT-R | tgaattactgaacacaacatTTTTAGTTTATGTATGTGTTTTTTG |  |
| GS-XKS1-XKS1t-F | atgttgtgttcagtaattcagagacagacaa |  |
| GS-XKS1-XKS1t-R | GCGGCCGCATGATTTAACAATAACCTAGCTCTTTCAATGCTC |  |
| GS-HO-R-F | TTGTTAAATCATGCGGCCGCCTGCAGacagtagctgacataccaagagatcttg |  |
| GS-HO-R-R | GCTCTAGAACTAGTGGATCCagagtttaagagaaaatgcgaaaatctggg |  |
| GS-HO-L-F | TCGAATTCCTGCAGCCCGGGGGATCCcttccatagcatctagcacatactcgat |  |
| OE-HHF1-K5-1-F | TCGAGGTCGACGGTATCGATGCGTTCTGAAAACTTCGCAT |  |
| OE-HHF1-K5-2-R | TTTACCACCACGACCTCTACCGGACATATTTTAC |  |
| OE-HHF1-K5-3-F | GTAGAGGTCGTGGTGGTAAAGGTCTAGGTAAAGG |  |
| OE-HHF1-K5-4-R | GCGGCCGCTCTAGAACTAGTACCGGGCAGTTGAATACGAA |  |
| OE-HHF2-K5-1-F | TCGAGGTCGACGGTATCGATCTGCGGTCACAAGAAGCAACG |  |
| OE-HHF2-K5-2-R | TTACCACCACGACCTCTACCGGACATTATTTTATTG |  |
| OE-HHF2-K5-3-F | GGTAGAGGTCGTGGTGGTAAAGGTCTAGGAAAAGGTG |  |
| OE-HHF2-K5-4-R | GCGGCCGCTCTAGAACTAGTACCGAAAAATGCATACATAAGGTTC |  |
| gRNA-HHF1-K5-F | GACTTTATGTCCGGTAGAGGTAAAGG |  |
| gRNA-HHF1-K5-R | AAACCCTTTACCTCTACCGGACATaa |  |
| gRNA-Y211-F | GACTTTGTAGAGGTAAAGGTGGTAAA |  |
| gRNA-Y211-R | AAACTTTACCACCTTTACCTCTACaa |  |
| OE-HHF1-K8R-1-F | TCGAATTCCTGCAGCCCGGGCGCGTTCTGAAAACTTCGCA |  |
| OE-HHF1-K8R-2-R | CCTAGACCACGACCACCTTTACCTCTACCGG |  |
| OE-HHF1-K8R-3-F | AAAGGTGGTCGTGGTCTAGGTAAAGGTGGTGC |  |
| OE-HHF1-K8R-4-R | GCGGCCGCTCTAGAACTAGTACCGGGCAGTTGAATACGAA |  |
| OE-HHF2-K8R-1-F | TCGAATTCCTGCAGCCCGGGCGCATTTGTATGGCAGGACG |  |
| OE-HHF2-K8R-2-R | CCTAGACCACGACCACCTTTACCTCTACCGG |  |
| OE-HHF2-K8R-3-F | AAAGGTGGTCGTGGTCTAGGAAAAGGTGGTGC |  |
| OE-HHF2-K8R-4-R | GCGGCCGCTCTAGAACTAGTCGGCAATGAAATGGAGGAGC |  |
| gRNA-HDA1-1-F | GACTTTGGAATAGGAGACTCATACTC |  |
| gRNA-HDA1-1-R | AAACGAGTATGAGTCTCCTATTCCaa |  |
| OE-P142-1-F | TCGAGGTCGACGGTATCGATCTCACTTGCCATTTTCCCGC |  |
| OE-P142-2-R | TCACATCTCATCGGCGTATTACAGTGCTCA |  |
| OE-P142-3-F | AATACGCCGATGAGATGTGATACGGACGGG |  |
| OE-P142-4-R | GCGGCCGCTCTAGAACTAGTTCCTCCAAAACCACTGCTCC |  |
| gRNA-HOS2-1-F | GACTTTGATTATACAACACTATACAC |  |
| gRNA-HOS2-1-R | AAACGTGTATAGTGTTGTATAATCaa |  |
| OE-P144-1-F | TCGAGGTCGACGGTATCGATCTCCAGCGGTTACCGATCTT |  |
| OE-P144-2-R | GGAGAAGCTTTGTACATTGGCGACACACCT |  |
| OE-P144-3-F | CCAATGTACAAAGCTTCTCCCGCAGCATTA |  |
| OE-P144-4-R | GCGGCCGCTCTAGAACTAGTTCCGCTGTGCATAAGGGTTA |  |
| gRNA-HST1-1-F | GACTTTGCAAGTCACACAAGATGCAG |  |
| gRNA-HST1-1-R | AAACCTGCATCTTGTGTGACTTGCaa |  |
| OE-P146-1-F | TCGAGGTCGACGGTATCGATAGAAGAGTCGCGCCTAAATGA |  |
| OE-P146-2-R | GTCAAGGGCGATGACGTGGCCAGTCTCGTA |  |
| OE-P146-3-F | GCCACGTCATCGCCCTTGACTAACCACCAA |  |
| OE-P146-4-R | GCGGCCGCTCTAGAACTAGTGTTGTCCAGCAAGGCCAAAG |  |
| gRNA-RPD3-1-F | GACTTTGCTGTCGTGTTACAGTGTGG |  |
| gRNA-RPD3-1-R | AAACCCACACTGTAACACGACAGCaa |  |
| OE-P148-1-F | TCGAGGTCGACGGTATCGATCGCAAATTCGTGCTCGACAA |  |
| OE-P148-2-R | CCTTCGTATCATACTATGAGCCGGGGAGGG |  |
| OE-P148-3-F | CTCATAGTATGATACGAAGGGTGGTTCGCA |  |
| OE-P148-4-R | GCGGCCGCTCTAGAACTAGTTTATCAACAGCGGTGGGACG |  |
| gRNA-ELP3-1-F | GACTTTGCAATGTGAGGACAACGATG |  |
| gRNA-ELP3-1-R | AAACCATCGTTGTCCTCACATTGCaa |  |
| OE-P150-1-F | TCGAGGTCGACGGTATCGATGTGCAATTGACCGAACGTGT |  |
| OE-P150-2-R | AGATGGCTCGAGACGGTCCATACATGTCGAA |  |
| OE-P150-3-F | TGGACCGTCTCGAGCCATCTTTGTCAGGGT |  |
| OE-P150-4-R | TAGAACTAGTCATGTACGGTCGCTTGAGGT |  |
| gRNA-GCN5-1-F | GACTTTGATTCTATTATTACAAGAAG |  |
| gRNA-GCN5-1-R | AAACCTTCTTGTAATAATAGAATCaa |  |
| OE-P152-1-F | TCGAGGTCGACGGTATCGATGCAGATCCTTCCTCAGTAGGC |  |
| OE-P152-2-R | CTCGCCGTACGGCTCACGGTCTTCCCTTAC |  |
| OE-P152-3-F | ACCGTGAGCCGTACGGCGAGACGATGTGAT |  |
| OE-P152-4-R | GCGGCCGCTCTAGAACTAGTCAGGCTCTGAAGGAGCACAA |  |
| gRNA-HPA2-1-F | GACTTTGCGAAGATAACATTACCGTA |  |
| gRNA-HPA2-1-R | AAACTACGGTAATGTTATCTTCGCaa |  |
| OE-P154-1-F | TCGAGGTCGACGGTATCGATGACAACATAGCATGCGGCTG |  |
| OE-P154-2-R | CACTTAATGTGGCTGTTTGCTTTCTTGGCA |  |
| OE-P154-3-F | GCAAACAGCCACATTAAGTGCTAGTGAGCGAGA |  |
| OE-P154-4-R | GCGGCCGCTCTAGAACTAGTCGGCACCGCTATCCTATGTT |  |
| gRNA-SAS3-1-F | GACTTTGTAGATACTGAATTATATGT |  |
| gRNA-SAS3-1-R | AAACACATATAATTCAGTATCTACaa |  |
| OE-P156-1-F | TCGAGGTCGACGGTATCGATCTCACTGCATCATTGGCGGT |  |
| OE-P156-2-R | TTATTCTTCGGGAAGAAGAGAGAGGGCGGT |  |
| OE-P156-3-F | CTCTTCTTCCCGAAGAATAAGCGGCGATGG |  |
| OE-P156-4-R | GCGGCCGCTCTAGAACTAGTCGAGGAGGAGGAGGATGACA |  |

**Table S2 gRNAs used in this work**

| gRNA ID | Sequence (5’-3’) |
| --- | --- |
| HO-gRNA | GACGACCAGGTCAGCTAGGG |
| HHF1-K5-gRNA | ATGTCCGGTAGAGGTAAAGG |
| HHF2-K5-gRNA | GGTAGAGGTAAAGGTGGTAA |
| HHF1-K8-gRNA | GGTAAAGGTGGTAAAGGTCT |
| HHF2-K8-gRNA | GGTAAAGGTGGTAAAGGTCT |
| HDA1-gRNA | GGAATAGGAGACTCATACTC |
| HOS2-gRNA | GATTATACAACACTATACAC |
| HST1-gRNA | GCAAGTCACACAAGATGCAG |
| RPD3-gRNA | GCTGTCGTGTTACAGTGTGG |
| ELP3-gRNA | GCAATGTGAGGACAACGATG |
| GCN5-gRNA | GATTCTATTATTACAAGAAG |
| HPA2-gRNA | GCGAAGATAACATTACCGTA |
| SAS3-gRNA | GTAGATACTGAATTATATGT |

**Figure S1**

**
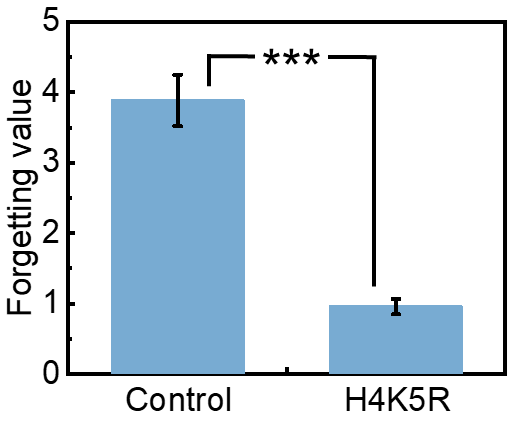
**

**Figure S1** Comparison of forgetting value between strain yYST210 (H4K5R) and control yYST12. The H4K5R and control yYST12 strains were incubated in synthetic complete-xylose medium (SX) continuously to induce XCM, and the strains were then transferred to synthetic complete-glucose medium (SG) for 0 and 8 days, respectively, and finally incubated in SX medium for 48 hours (h). ***P<0.001
